# Notochordal cell conditioned medium (NCCM) regenerates end-stage human osteoarthritic articular chondrocytes and promotes a healthy phenotype

**DOI:** 10.1186/s13075-016-1026-x

**Published:** 2016-06-02

**Authors:** Sebastian Müller, Lina Acevedo, Xiaomei Wang, M. Zia Karim, Ajay Matta, Arne Mehrkens, Stefan Schaeren, Sandra Feliciano, Marcel Jakob, Ivan Martin, Andrea Barbero, W. Mark Erwin

**Affiliations:** Department of Orthopaedics and Traumatology, University Hospital Basel, Spitalstrasse 21, 4031 Basel, Switzerland; Department of Biomedicine, University Hospital Basel, University of Basel, Hebelstrasse 20, 4031 Basel, Switzerland; Krembil Research Institute, Toronto Western Hospital, Divisions of Neurological and Orthopaedic Surgery, University of Toronto, 60 Leonard Street, KDT5-407, Toronto, ON M5K 1K2 Canada; Department of Spine Surgery, University Hospital Basel, Spitalstrasse 21, 4031 Basel, Switzerland

**Keywords:** Notochordal cell conditioned medium, OA chondrocytes, Cartilage, Regenerative medicine, OA treatment

## Abstract

**Background:**

Notochordal cell conditioned medium (NCCM) derived from non-chondrodystrophic dogs has pro-anabolic and anti-catabolic effects upon nucleus pulposus (NP) cells. Here, for the first time, we assessed the ability of NCCM to influence the production of extracellular matrix and inflammatory proteins by healthy and osteoarthritic human chondrocytes within engineered cartilage tissues. We hypothesized that, similar to its action on NP cells, NCCM exerts metabolic and anti-catabolic effects on human articular chondrocytes and has the potential to significantly counteract inflammatory mediators.

**Methods:**

Chondrocytes from nine non-osteoarthritic patients and from six osteoarthritic (OA) donors at the time of total knee arthroplasty were chondro-differentiated in pellets for 2 weeks. Non-OA pellets were exposed for 72 hours to IL-1β/TNF-α and then cultured up to 14 days in 2 % FBS-supplemented NCCM or 2 % FBS-supplemented medium (control (ctr)). OA pellets were cultured in NCCM or ctr medium without pro-inflammatory treatment. Tissues after each culture phase were analyzed biochemically (GAG/DNA), (immuno-) histologically (collagen I, II and GAG) and by Western blotting. Supernatants were analyzed by ELISA.

**Results:**

Response to NCCM was age and disease dependent with healthy chondrocyte pellets (from donors >55 years of age) recovering their glycosaminoglycan (GAG) contents to baseline levels only with NCCM. OA pellets treated with NCCM significantly increased GAG content (1.8-fold) and levels of hyaluronic acid link protein (HAPLN), fibromodulin and SOX-9. The catabolic proteins (matrix metalloproteinase (MMP)-3 and MMP-13) and pro-inflammatory enzyme levels (cyclooxygenase-2 (COX-2)) were markedly reduced and there was significantly reduced secretion of pro-inflammatory chemokines (IL-6 and IL-8).

**Conclusions:**

NCCM restores cartilage matrix production of end-stage human OA chondrocytes towards a healthy phenotype and suppresses the production of inflammatory mediators. Harnessing the necessary and sufficient factors within NCCM that confers chondroprotection and regenerative effects could lead to a minimally invasive agent for treatment of degenerative and inflammatory joint diseases.

**Electronic supplementary material:**

The online version of this article (doi:10.1186/s13075-016-1026-x) contains supplementary material, which is available to authorized users.

## Background

Osteoarthritis (OA) is not only the most common joint disease worldwide causing pain and functional limitations, but it can also lead to worsening of co-morbidities, such as diabetes and cardiovascular disease, due to reduced activity [1, 2]. OA, in particular of the knee joint, already generates annual costs of US$185 billion in the USA and due to demographic changes the costs associated with OA are predicted to increase in the future, necessitating the development of novel, disease-modifying interventions [3–5].

Beyond physical therapy modalities, anti-inflammatory medications, and intra-articular injections there are no other effective treatments currently available for OA apart from (for end-stage OA) joint arthroplasty. The quest for an effective disease-modifying osteoarthritic agent/drug (DMOAD) is an area of intense investigation worldwide that could be enabled by studying degenerative joint diseases sharing many features with OA, such as degenerative disc disease (DDD). DDD, like OA, involves an avascular, ischemic and hypoxic niche, the loss of extracellular matrix homeostasis, progressive cell death, and the development of a pro-catabolic, pro-inflammatory state with impaired cellular repair and known propensity for chronic pain [6].

Adult nucleus pulposus (NP) cells are commonly referred to as “chondrocyte-like cells”, given their similarities in extracellular matrix (ECM) production, cell size, and the hypoxic, ischemic environment within which they reside, even though there are differences in the expression of certain ECM molecules [7, 8]. Consistent with its participation in development, cartilage and the notochord express a number of similar genes as those that encode type II and type IX collagen, aggrecan, SOX-9 and chondromodulin, suggesting that these cells may have conserved common physiological properties [8–12]. These common features, taken together with previous work, suggest that the soluble factors secreted by notochordal cells may also confer anabolic and anti-catabolic effects upon cartilage.

Within the spectrum of DDD, the peculiar phenomenon of the non-chondrodystrophic canine (NCD) is of particular importance to possible treatments for OA. The NCD animal does not develop DDD until much later in life, if at all, ostensibly due to the persistence of notochordal cells within the NP of the intervertebral disc (IVD) [13, 14]. In contrast, it is widely known that humans (like chondrodystrophic beagles and dachshunds) lose their notochordal cells early in life and are far more prone to developing DDD [15, 16]. A number of publications detailing the mechanism of action of notochordal cells have established that these cells provide an anti-degenerative effect upon cells within the NP, including anti-apoptotic effects [17–19]. Furthermore, notochordal cell conditioned medium (NCCM) has been shown to induce an upregulation of anabolic/matrix protective genes such as type II collagen, the CD44 receptor and TIMP-1, as well as downregulation of matrix-degrading genes like matrix metalloproteinase-3 (MMP-3) [18, 20, 21]. Furthermore, NCCM treatment increases cell proliferation and proteoglycan production, and directs degenerative IVD NP cells towards healthier phenotypes [22, 23]. Additionally, mesenchymal stem cells (MSC) treated with NCCM produce more proteoglycan compared to control medium [23, 24].

We hypothesized that, similar to its action on NP cells, NCCM exerts metabolic and anti-catabolic effects on articular chondrocytes. We were interested in the possibility that soluble factors contained within NCCM could be exploited in a chondroprotective strategy for the treatment of osteoarthritis/cartilage injury. Therefore, we elected to use NCCM obtained from NCD dogs to treat human chondrocytes conditioned with pro-inflammatory cytokines or chondrocytes obtained at the time of total knee arthroplasty in a proof-of-principle study. If effective, recombinant forms of the necessary and sufficient factors contained within NCCM could be used in a minimally invasive strategy. We have demonstrated previously that there is considerable preservation in genomic and proteomic sequences between canine and human species in that we have determined the presence of connective tissue growth factor (CTGF) within NCCM in our first proteomic analysis of NCCM, validating that the use of cross-species conditioned medium is a valid approach [21].

## Methods

### Preparation of notochordal cell conditioned medium (NCCM)

We have reported on the use of NCCM extensively in our past publications [17, 21, 25]. In the current study, notochordal cell-rich IVD NPs were obtained from 10 different NCD dogs within an age range of 12–18 months in collaboration with a licensed animal facility and all practices were in accordance with the animal care policies and ethics approval board of The University Health Network, Toronto, Ontario, Canada. Briefly, after humane euthanasia, the T6-L6 spinal segments were removed aseptically *en bloc* and a wide laminectomy performed separating the posterior elements from the vertebral bodies. Next, we removed the NPs in accordance with our established methods and developed NCCM by culturing the NPs in advanced Dulbeccos’s modified Eagle’s medium F/12 (ADMEM/F12) supplemented with 2 % fetal bovine serum (FBS) (v/v) and penicillin/streptomycin (PS) (100 U/mL) [18]. We collected the NCCM at 24-hour intervals for 5 days and at each interval the NCCM was centrifuged for 20 minutes at 3000 g and filtered using 0.2-μm filters (Corning, USA). Next the media were transferred to 15-mL aliquots and frozen at –80̊ C. Consistent with our established methods once the media were collected, filtered and centrifuged they were pooled in order to remove any batch-to-batch variability and all culture conditions were performed with identical conditioned medium. We generated 2 % FBS (v/v)-supplemented ADMEM/F12 as the control medium (ctr).

### Healthy chondrocyte culture

#### Cartilage sample collection, and cell isolation and expansion

In the first experiments cartilage tissue was harvested from nine donors (18–68 years of age), from macroscopically healthy-looking areas of their knee joints. Two samples were taken from an 18-year-old man and a 68-year-old man during diagnostic arthroscopy and seven samples were taken from cadavers within 24 hours after donors had deceased (age range 41–64 years). Either patients or relatives gave informed consent for tissue harvest. All tissue samples were minced with a scalpel into small pieces that were then digested overnight in 0.2 % collagenase II (300 U/mg, Worthington Biochemical Corp, Lakewood, NJ, USA) in an orbital shaker at 37 °C. The isolated chondrocytes were then expanded for two passages in basal medium (BM, DMEM, 10 mM HEPES, 1 mM sodium pyruvate, 100 U/ml penicillin, 100 μg/mL streptomycin, and 0.29 mg/mL glutamate (all from Invitrogen)) supplemented with 10 % FBS, 5 ng/mL fibroblast growth factor-2 (FGF-2) and 1 ng/mL transforming growth factor (TGF)ß1 in a humidified incubator (37 °C, 5 % CO_2_, 19 % oxygen) as previously described [26].

##### Chondrogenic differentiation in pellets

Chondrogenic differentiation was induced by culturing the expanded chondrocytes in pellets using defined serum-free medium as previously described [27]. Briefly, cells were re-suspended in chondrogenic medium (BM, 1.25 mg/mL human serum albumin, ITS-A (Invitrogen), 10 ng/mL TGF-ß1 (R&D Systems), and 10^-7^ M dexamethasone and 0.1 mM ascorbic acid 2-phosphate (Sigma-Aldrich). Aliquots of 2.5 × 10^5^ cells/250 μL were centrifuged at 250 g for 5 minutes in 1.5 mL screw-cap Eppendorf tubes. Pellets were cultured for 2 weeks in a humidified incubator (37 °C, 5 % CO_2_, 19 % oxygen) with a change of medium twice a week.

##### Inflammation and recovery cultures

**Fig. 1:**
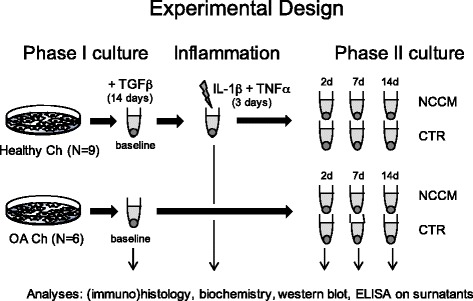
Outline of experimental approach. Human articular chondrocytes (*Ch*) collected from healthy and osteoarthritic (*OA*) cartilage tissues were expanded in vitro and then cultured in pellets (phase I culture). Resulting tissues were then exposed to interleukin 1 beta (IL-1β) and tumor necrosis factor-alpha (TNF-α) for 3 days (*d*) and then cultured for an additional 2 weeks (phase II culture) in medium containing 2 % FBS-supplemented notochordal cell conditioned medium (*NCCM*) or in control medium (2 % FBS-supplemented ADMEM/F12, (*CTR*)). Pellets generated from OA chondrocytes were cultured in NCCM or CTR medium without any pro-inflammatory treatment. *TGF* transforming growth factor

After 2 weeks of culture, the pellets were harvested for baseline histological and biochemical assessments, while the remaining were exposed to IL-1β (Sigma, I9401) and TNF-α (Peprotech), each at 1 ng/mL, for 72 hours. During this inflammatory phase, pellets were cultured in chondrogenic medium deprived of TGF-β1 and dexamethasone. Pellets were then harvested for analysis or cultured for 2, 7, and 14 days in NCCM or 2 % FBS-supplemented ADMEM/F12 (ctr) (Fig. 1).

To assess possible age-dependent differences in the response of chondrocytes to the inflammatory factors and post-inflammatory NCCM treatment, donors were assigned to two arbitrary age groups: group 1, ≤55 years of age (*n* = 4) and group 2, >55 years of age (*n* = 5).

### Osteoarthritic chondrocyte culture

Based on the findings generated with the healthy chondrocytes, in a second experiment we investigated the effects of NCCM on chondrocytes obtained from six patients (ages 60–82 years, all female), who suffered from end-stage OA and were undergoing total knee arthroplasty. Within 2 hours of tissue harvest, pieces of grossly visible degraded osteoarthritic cartilage were collected and directly processed as described above. Isolated OA chondrocytes were also expanded and chondrogenically cultured as described for the healthy chondrocytes. After 2 weeks of chondrogenic differentiation, baseline pellets were collected for histological, immunohistochemical and biochemical analyses. Hereafter, the remaining pellets were directly cultured in NCCM or control medium. Medium was changed twice a week and pellets were harvested at 2, 7, and 14 days (see Fig. 1 for the experimental plan). Culture medium was also collected from baseline pellets and pellets cultured in control and NCCM medium, to quantify the amounts of released cytokines.

In order to assess whether the soluble glycosaminoglycans (GAGs) contained in NCCM could have served as active factors, chondrogenic pellets obtained from one of the NCCM-responder donors were also cultured with control medium supplemented with 1 mg/mL chondroitin sulphate (Sigma, CS9819) for 2 weeks; the amount of chondroitin sulphate used corresponded to the concentration of GAG measured in the basal NCCM medium used in this study).

### Analytical methods

#### Biochemical analysis

To measure the amounts of sulfated GAG and DNA, pellets (*n* = 3) from each group and condition were digested with Proteinase K (0.5 ml of 1 mg/mL protease K in 50 mM Tris with 1 mM EDTA, 1 mM iodoacetamide, and 10 mg/mL pepstatin-A) for 15 hours at 56 °C. The GAG content was measured spectrophotometrically after reaction with dimethylmethylene blue and chondroitin sulfate as a standard (Sigma, C8529), using the Blyscan color assay kit (Biocolor Ltd, Carrickfergus, UK). For further analysis the mean amount of GAG for each condition was normalized to the corresponding mean amount of DNA, which was measured spectrofluorometrically using the CyQUANT cell proliferation assay kit (Molecular Probes, Eugene, OR, USA), with bovine DNA as a standard. GAG and DNA content is reported as μg GAG/μg DNA for samples and supernatants or normalized to baseline values, respectively. Additionally, GAG was also quantified in the basal NCCM medium.

#### Histologic and immunohistochemical analysis

Pellets (*n* = 2) for each condition were fixed in 4 % formalin for 24 hours, washed with PBS, and then embedded in paraffin blocks. For (immuno-) histological staining the samples were sectioned at a thickness of 5 μm. Sections were then stained with Safranin O for sulfated GAGs or processed for more detailed immunohistochemical analyses with antibodies against collagen type I collagen (mouse-anti-human, Clone I-8H5, MP Biomedicals) and type II collagen (mouse-anti-human, clone II-4 CII, MP Biomedicals, Santa Ana, CA, USA).

#### Western blotting

The expression of anabolic (SOX-9, HAPLN1, and fibromodulin), and catabolic proteins (MMP-3, MMP-13, and COX-2) in either NCCM-treated or Ctr-treated pellets was detected by western blot with β-actin used as a loading control. Total protein was extracted from OA pellets of every donor for each condition. We used the Pierce BCA Protein Assay Kit (Thermo Scientific, Rockwood, IL, USA) together with radioimmunoprecipitation assay (RIPA) lysis buffer for protein extraction and measurement of the protein concentration according to the manufacturer’s instructions. Results were then read with a Perkin Elmer Victor3 Multilabel Counter (model 1420). Proteins were resolved on 10 % sodium dodecyl sulphate-polyacrylamide gels (SDS-PAGE) and then electro-transferred onto polyvinyledendifluroide (PVDF) membranes (BioRad, Hercules, CA, USA). SDS-PAGE gels were then transferred to nitrocellulose membrane in transfer buffer (BioRad) containing 20 % methanol. Membranes were washed with TBS-T (TBS, 0.1 M, pH = 7.4; 0.1 % Tween 20, Bioshop) and then blocked with 5 % non-fat milk extract in a TBS blocking buffer for 1 hour in an orbital shaker at room temperature. We then incubated the membranes with the primary antibody of each protein of interest (see Additional file 1). The next morning, membranes were washed three times with Tween 20 (0.1 %)-TBS-T for 5 minutes each in an orbital shaker and thereafter, incubated with goat anti-rabbit IgG (H + L)-HRP conjugate (Bio-Rad cat. 170-6515), goat anti-mouse IgG(H + L)-HRP conjugate (Bio-Rad cat. 170-6516) and rabbit anti-goat F(AB)2 HRP XADs (NOVEX cat. A24452) secondary antibodies, respectively, diluted at an appropriate dilution in 1 % non-fat powdered milk for 1 hour at room temperature. Conjugates were matched with the appropriate antibody, i.e., rabbit polyclonal anti-Sox9 (Abcam cat. ab71762) 1:200 was matched with goat anti-rabbit IgG conjugate, etc. Blots were then washed three times with Tween 20 (0.1 %)-TBS. Signals were visualized by exposing the membranes to enhanced chemiluminescence (ECL) reagent (Biorad) according to the manufacturer’s instructions. Densitometry values were calculated for each band using ImageJ (National Institute of Health). Results of western blot analysis are presented as fold-change for pellets after 14 days treatment (NCCM or Ctr) compared to baseline.

### Enzyme-linked immunosorbent assay

We determined differential OA chondrocyte pellet cytokine secretion using supernatants obtained from two representative responder donors using the multi-analyte cytokine profiling ELISA kit according to the manufacturer’s instruction (Qiagen, ML, USA) evaluating the expression of IL-6 and IL-8 in particular. We performed each experiment in triplicate.

### Statistical analysis

Statistical evaluation was performed using SPSS 21 (IBM, Armonk, NY, USA). Values are presented as mean with corresponding standard deviation (SD) or as normalized to baseline values. Differences between experimental groups were assessed using the Mann-Whitney *U* test for non-parametric samples. *P* values <0.05 were considered significant.

## Results

### NCCM restores post-inflammation GAG production by healthy articular chondrocytes only from older donors

**Fig. 2:**
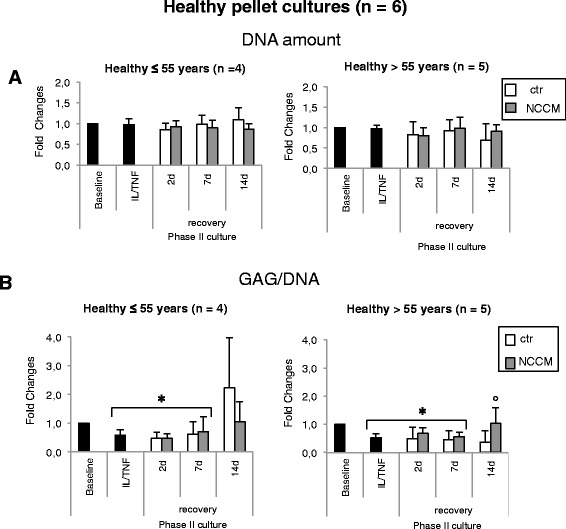
Biochemical analyses of pellets generated with healthy chondrocytes. **a** DNA and **b** sulfated glycosaminoglycan (GAG) content of chondrocyte pellets normalized to the DNA content (GAG/DNA). Data are expressed as fold-change in patients ≤55 years of age (*left*) or >55 years of age (*right*). All pellets were treated after the phase I culture (baseline) with the inflammatory treatment (IL-1β + TNF) and after 2, 7, and 14 additional days of culture in control (*ctr*) medium or notochordal cell conditioned medium (*NCCM*) (see Fig. 1 for experimental design). Data are means ± SD (*n* indicates the number of cartilage donors; for each donor two to three experimental replicates were analyzed); *p < 0.05 from baseline; °*p* < 0.05 from ctr (same recovery time). Treatment with NCCM does not result in any significant increase in DNA in either group of patients (**a**). In chondrocyte pellets from patients >55 years of age, NCCM treatment results in a significant increase in GAG/DNA content, returning to baseline levels at 14 days of culture; °*p* < 0.05 (**b**)

Cells from the two age groups (group 1 ≤55 years, group 2 >55 years of age) proliferated at a similar rate (number of doublings/day 0.52 ± 0.12 and 0.45 ± 0.08, respectively; *p* > 0.05). The DNA content of pellets generated from cells of individuals within the two age groups did not significantly vary after the treatment with inflammatory factors or during the subsequent phase II culture in control or NCCM-medium (Fig. 2a). However, the GAG/DNA content of the pellets decreased significantly following IL-1β and TNF-α exposure (by 1.7-fold and 1.9-fold, respectively) for group 1 (≤55 years of age), and group 2 (>55 years of age). The extent of GAG recovery varied in the two groups. In group 1 the GAG/DNA content of pellets remained lower (as compared to baseline) beginning on day 2 of phase II culture, with modest recovery but still below baseline by day 7; however, it did not increase significantly by day 14 (Fig. 2b). There were no significant differences in GAG/DNA content in Ctr-treated or NCCM-treated pellets during any of the phase II cultures (Fig. 1b (≤55 years of age)). On the other hand, although pellets treated with control medium in group 2 (>55 years of age) also had lower GAG/DNA content for the entire phase II culture (after treatment with pro-inflammatory mediators) (Fig. 2b), by day 14 the GAG/DNA content of NCCM-treated pellets had increased significantly to return to baseline levels (Fig. 2b).

These initial results indicate that while chondrocyte pellets from young individuals have an intrinsic capacity to recover following an inflammatory insult, NCCM confers such recovery upon tissue pellets of chondrocytes from older donors. As the articular cartilage from patients over the age of 60 years, even if appearing macroscopically healthy, could present pre-osteoarthritic changes, we chose to investigate the ability of NCCM to rescue articular chondrocytes obtained from patients with end-stage OA.

### NCCM upregulates cartilage matrix production in OA chondrocytes

**Fig. 3:**
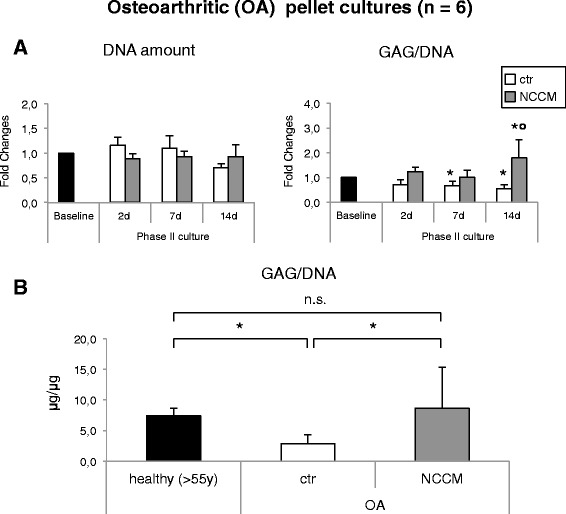
Biochemical analyses of pellets generated with osteoarthritic (*OA*) chondrocytes. **a** DNA contents (*left*) and sulfated glycosaminoglycan (*GAG*) contents normalized to the DNA content (GAG/DNA) (*right*) of pellets after the phase I culture (baseline) and after 2, 7, and 14 additional days of culture in control (*ctr*) medium or notochordal cell conditioned medium (*NCCM*) (see Fig. 1 for the experimental design). Levels are expressed as fold-difference from those measured at baseline. Data are means ± SD (*n* indicates the number of cartilage donors; for each donor two to three experimental replicates were analyzed); *p < 0.05 from baseline; °*p* < 0.05 from ctr (same culture time). **b** GAG/DNA content of pellets generated by healthy chondrocytes at baseline (*n* = 4, same as those in Fig. 2, > 55 years of age) and OA chondrocytes after phase II culture in ctr medium and NCCM; *significant difference at *p* < 0.05; *n.s.* difference not significant

After 14 days of pellet culture the DNA content of pellets in the Ctr group was lower (1.4-fold, *p* < 0.05) than the baseline content and remained comparable to baseline levels in pellets cultured with NCCM at all the investigated time points (Fig. 3a). The GAG/DNA content of Ctr-cultured pellets slightly decreased over time and remained largely constant, such that at the last time point it was 1.8-fold lower than at baseline. In contrast, OA chondrocyte pellet cultures treated with NCCM had drastically enhanced accumulation of GAG/DNA, such that by day 14, the accumulated GAG/DNA content was 1.8-fold higher (*p* < 0.05) than at baseline (Fig. 3a).

We thus compared the GAG/DNA content of pellets generated by healthy chondrocytes (from donors > 55 years) with those of pellets generated from OA chondrocytes. At the end of culture phase II, OA chondrocytes cultured with NCCM accumulated GAG at a level comparable to that of baseline healthy chondrocytes, whereas the GAG content of Ctr-cultured pellets was instead 2.6-fold lower (*p* < 0.05) (Fig. 3b).

**Fig. 4:**
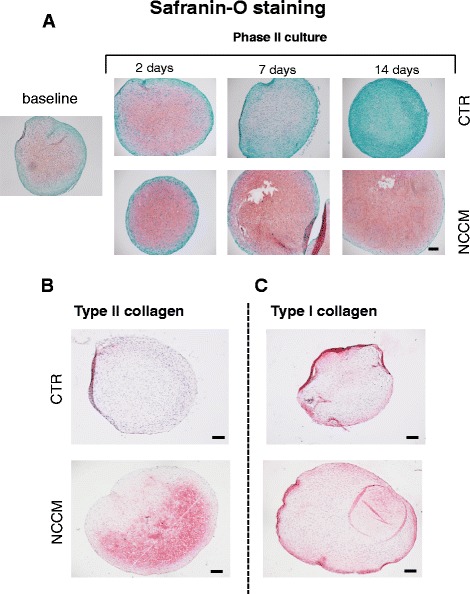
Histological and immunohistochemical characterizations of pellets generated by osteoarthritic chondrocytes. **a** Safranin-O staining of baseline pellets and pellets after 2, 7, and 14 additional days of culture in control (*CTR*) medium or notochordal cell conditioned medium (*NCCM*). There is a clear increase in Safranin-O with NCCM treatment at 14 days of culture. Collagen type II (**b**) and collagen type I (**c**) immunostochemical staining of representative pellets after 14 days phase II culture in CTR medium or NCCM (see Fig. 1 for the experimental design). *Bars* = 100 μm. As with the Safranin-O staining, 14 days of NCCM culture induces a marked increase in collagen type II staining with no difference in type I collagen

Histological analyses confirmed the biochemical results. The intensity of Safranin-O and type II collagen staining decreased in pellets after 7 days of culture in Ctr medium and become almost undetectable after 14 days. However, Safranin-O and collagen type II staining in pellets cultured for 14 days with NCCM were higher than those at baseline (Fig. 4).

### The anabolic effects of NCCM are not mediated by chondroitin sulfate

We wondered if the aforementioned anabolic effects of NCCM were merely due to the chondroitin sulfate (CS) contained within the NCCM. Therefore, we exposed chondrocyte pellets obtained from one of the NCCM-responsive OA donors (82 years of age, female) to 1 mg/mL CS (i.e., equivalent CS concentration as in the NCCM used in the previous experiments) for 14 days and assessed them biochemically. The GAG/DNA content of pellets cultured for 14 days in CS decreased 2.5-fold compared to baseline, whereas the GAG/DNA content increased in pellets cultured with NCCM (Table 1).

**Table 1:** Comparison of GAG/DNA accumulation within pellets treated with CTR, Chondroitinsulfate or NCCM medium

	GAG/DNA pellets (μg/μg)
Baseline	12.1 ± 1.4
14 days of CTR	3.7 ± 1.9
14 days of CS	4.8 ± 0.3
14 days of NCCM	13.4 ± 5.8

Values are mean ± SD. Control (CTR) media comprised 2 % FBS-supplemented advanced DMEM/F-12. The chondroitin sulfate (CS) medium comprised Ctr medium supplemented with 1 mg/mL CS. NCCM was supplemented with 2 % FBS. The accumulation of GAG/DNA in pellets treated with NCCM was clearly superior to CTR and CS, demonstrating that the effects of NCCM are not due to CS. *GAG* glycosaminoglycan

### NCCM enhances the production of anabolic factors and reduces the production of catabolic mediators in OA chondrocytes

**Fig. 5:**
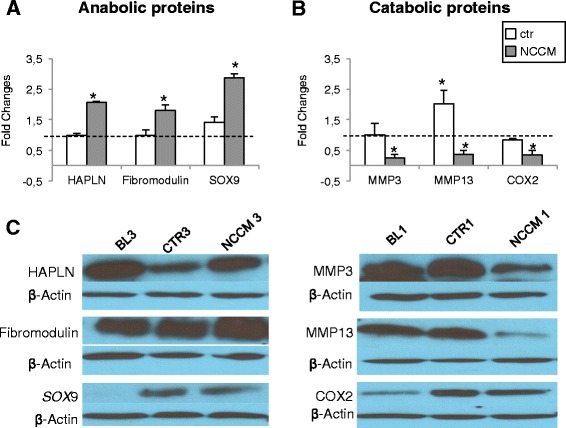
Western blot analyses of pellets generated by osteoarthritic (OA) chondrocytes. Data representative of five independent samples of notochordal cell conditioned medium (*NCCM*)-treated human OA chondrocyte pellets at 14 days of culture compared to controls (*CTR*). Data are fold-changes normalized to baseline values. **a** Normalized expression of anabolic proteins. **b** Normalized expression of catabolic proteins. Representative blots (one of five samples) are shown (**c**). NCCM significantly increases the levels of anabolic extracellular matrix proteins and the master chrondrogenic regulator sex determining region Y box 9 (*SOX9*), compared to CTR treatment; *p < 0.05). NCCM significantly decreases the levels of the matrix-degrading matrix metalloproteinase-3 (*MMP-3*) and MMP-13, and the pro-inflammatory enzyme cyclooxygenase-2 (*COX2*); *p < 0.05. *HAPLN* hyaluronic acid link protein

We analyzed NCCM and control-cultured pellets from OA chondrocytes using western blotting methods to quantitatively assess differences in proteins that are important in cartilage anabolism/homeostasis and catabolism/degradation. NCCM significantly enhanced the levels of HAPLN1, fibromodulin and the master chrondrogenic regulator *SOX*-9 (2.1-fold, 1.8-fold, and 2.9-fold higher, respectively, after NCCM treatment vs baseline) but remained unchanged in the Ctr group (Fig. 5a). Furthermore, NCCM significantly downregulated the major catabolic/degradative mediators MMP-3, MMP-13, and COX-2 (4.2-fold, 2.8-fold, and 2.7-fold, respectively) (Fig. 5b). On the other hand, in control cultures MMP-3 and COX-2 expression remained largely at baseline levels, whereas MMP-13 increased (2.0-fold, *p* < 0.05) in Ctr-cultured pellets (Fig. 5b). Representative western blots are shown in Fig. 5c).

### NCCM-treated chondrocyte pellets decrease the secretion of pro-inflammatory cytokines

**Fig. 6:**
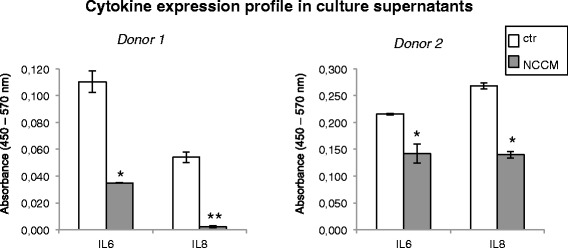
Cytokine ELISA of supernatants from pellets generated by osteoarthritic chondrocytes. These data indicate that notochordal cell conditioned medium (NCCM)-treated chondrocyte pellet cultures secrete significantly less IL-6 and IL-8 than control (*ctr*) cultures (*donor 1*, female, 73 years of age; *donor 2*, female, 82 years of age); *p < 0.05 compared to ctr; **p < 0.01 compared to ctr

We evaluated the secretion of a number of cytokines by pellet cultures obtained from cells of two patients with OA (donor 1, female, 73 years of age and donor 2, female, 82 years of age) after 14 days of phase II culture in NCCM and control medium using the SABiosciences multi-analyte cytokine ELISA SABiosciences kit (Qiagen, ML, USA). We found that in both cases, treatment with NCCM significantly reduced the secretion of IL-6 and IL-8 in pellet cultures obtained from end-stage OA cartilage, whereas control treatment did not have any effect (Fig. 6).

## Discussion

Here we report for the first time, that NCCM has a chondroprotective/regenerative effect upon human articular chondrocytes and that these effects were age and disease dependent. NCCM treatment restored OA chondrocyte cartilage matrix production to healthy chondrocyte baseline levels, significantly counteracted the production of potent pro-inflammatory cytokines, and suppressed the secretion of key pro-inflammatory cytokines.

Osteoarthritis is a multi-factorial disease process driven by the master regulators of cellular/ECM destruction, IL-1β and TNFα, pro-inflammatory molecules such as the COX-2-dependent cartilage-destructive enzyme prostaglandin E_2_ (PGE2), IL-6, IL-8, and the ECM-degrading enzymes such as the MMPs. Increased levels of PGE_2_ act in a positive feedback loop to promote the synthesis of IL-1β, TNFα, IL-6, and IL-8, resulting in a self-propelling autocrine/paracrine-mediated inflammatory process [28]. The action of the inflammatory cytokines such as IL-1β, IL-6, and IL-8 decreases the synthesis of vital ECM molecules such as proteoglycans and type II collagen, thereby diminishing the hydrophilic properties of the ECM leading to further degradation [28–31]. It has been shown that IL-6 correlates with worsening osteoarthritic lesions on radiographs and that IL-6 and IL-8 potentiate the pro-inflammatory action of IL-1β [32]. Similarly, it has been shown that bulging/herniated IVDs express high levels of these pro-inflammatory cytokines and that higher levels of IL-6 and IL-8 within these tissues correlate with increasing back pain suggesting that these two cytokines share commonality in disease progression and underlying symptomatology [32].

An effective biologic therapy to treat OA would necessarily need to inhibit catabolism, increase anabolism, and interfere with the pro-inflammatory milieu present in the target tissue. Contemporary approaches to treating OA employ orally administered analgesic or anti-inflammatory medication and/or intra-articular injections (usually of a steroid combination) for single joints [33]. The injection of hyaluronic acid and platelet-rich plasma (PRP) has also been attempted; however, the results have been rather disappointing as such treatment only provided short-term symptom relief, if at all [34–37]. However, to date, no intervention has been found to have the ability to influence the biology of degenerative joint disease, and thus, the notion of chondroprotection, whereby factors delivered to cartilage that may mediate its degeneration, is an attractive therapeutic goal and would be a major breakthrough in the treatment of OA.

To this end, lessons learned with respect to notochordal cell interaction within the IVD could be applied to the development of biologically based anti-OA therapeutic agents. We (WME and AM) have previously reported that NCCM confers anti-apoptotic, pro-anabolic and anti-catabolic effects upon IVD NP cells [17–20]. Although we do not report apoptotic signaling here, it is clear that NCCM antagonizes the inflammatory cascade, suppresses catabolism, and confers anabolic effects upon vital ECM molecules. It may be that by suppressing the levels of pro-inflammatory cytokines and enzymes, NCCM might enable chondrocytes to restore homeostatic regulation. Interestingly we noted a more robust response to NCCM by osteoarthric vs healthy chondrocytes. While the effects of NCCM might be more effective within the inflammatory milieu, it could be that in addition to its anti-inflammatory activity, the bioactive factors within NCCM could also target specific subpopulations of chondrogenic progenitor cells known to be present with advancing OA [38, 39]. The drug Diacerein has been reported to antagonize pro-inflammatory changes in chondrocytes in vitro through the suppression of MMP1 and IL-8 activity, suggesting its potential utility as a treatment for OA [30]. However, there is no evidence that this drug is capable of inducing the broad range of anti-catabolic and pro-anabolic effects necessary for an effective anti-OA intervention. This is in contrast to the chondroprotective and regenerative effects conferred upon human chondrocytes by NCCM in our study, including the re-differentiation of end-stage OA chondrocytes to a healthy phenotype.

As with all investigations, there are some limitations to this study. For example, we studied the response of cultured engineered cartilaginous tissue to treatment with NCCM but it might be that cells within a native tissue may exhibit different responses. However, we preferred to use this in vitro method because it allows better calculation of new matrix deposition as compared to the organ culture model. It is important to illustrate that the OA cartilage obtained in this study was obtained from joints that had not been injected with steroids for at least 4–6 months, providing a robust washout period that would account for any transient changes in response to treatment. Even so, all samples were subjected to the same no-treatment controls and the responses for all donor cells were very consistent, dismissing the likelihood of biological variability associated with any exogenous intervention.

## Conclusions

NCCM treatment restored OA chondrocyte cartilage matrix production to healthy chondrocyte baseline levels, significantly counteracts the production of potent pro-inflammatory cytokines, suppressed the secretion of key pro-inflammatory cytokines and upregulated the master chondrogenic regulator *SOX9*. The precise signaling mechanisms whereby NCCM confers pro-anabolic, anti-catabolic, and anti-inflammatory effects remain to be determined. However, these novel observations open the door to the development of an entirely novel molecular therapy for the treatment of osteoarthritis and chondroprotection, utilizing the necessary and sufficient factors secreted by the notochordal cell-rich IVD NP.

## Abbreviations

ADMEM/F12, advanced Dulbeccos’s modified Eagle’s medium F/12; BM, basal medium; COX-2, cyclooxygenase-2; CS, chondroitine sulphate; CTGF, connective tissue growth factor; Ctr, control; DDD, degenerative disc disease; DMOAD, disease-modifying osteoarthritic agent/drug; ECL, enhanced chemiluminescence; ECM, extracellular matrix; ELISA, enzyme-linked immunosorbent assay; FBS, fetal bovine serum; FGF, fibroblast growth factor; GAG: glycosaminoglycan; HAPLN, hyaluronic acid link protein; HRP, horseradish peroxidase; IL, interleukin; IVD, intervertebral disc; MMP, matrix metalloproteinase; MSC, mesenchymal stem cells; OA, osteoarthritis; NCCM, notochordal cell conditioned medium; NCD, non-chondrodystrophic; NP, nucleus pulposus; PBS, phosphate-buffered saline; PRP, platelet-rich plasma; PS, penicillin streptomycin; PVDF, polyvinyledendifluroide; SDS-PAGE, sodium dodecyl sulphate-polyacrylamide; TBS-T, Tween Tris-buffer saline; TIMP, tissue inhibitor of metalloproteinase; TNF, tumor necrosis factor

## Additional file

Additional file 1: Supplemental material: antibodies used for western blot analysis. (DOCX 36 kb)
