# Supplementary material for: Notochordal cell conditioned medium (NCCM) regenerates end-stage human osteoarthritic articular chondrocytes and promotes a healthy phenotype
Source: Arthritis Res Ther. 2016 Jun 2;18:125. doi: 10.1186/s13075-016-1026-x (PMC4890286; doi:10.1186/s13075-016-1026-x)
Supplement: Additional file 1: — Supplemental material: antibodies used for western blot analysis. (DOCX 36 kb) [file 13075_2016_1026_MOESM1_ESM.docx]

**Supplemental material:**

**Antibodies used for Western Blot analysis**

**List of primary Antibodies** (all incubated in a 1% TBS-Milk solution at 4°C overnight):

*SOX-9*: rabbit polyclonal anti sox9 (Abcam cat. ab71762) 1:200 dilution

*HAPLN1(K-14):* goat polyclonal anti HAPLN1(K-14) (Santa Cruz Biotechnology cat. sc-46862) 1:500 dilution

*FIBROMODULIN*: rabbit polyclonal anti-Fibromodulin (NOVUS cat. NBP216494) 1:200 dilution

*MMP3*: rabbit monoclonal anti MMP3 (Abcam cat. ab52915) 1:200 dilution

*MMP13*: rabbit polyclonal anti MMP13 (Abcam cat. ab39012) 1:200 dilution

COX2: rabbit polyclonal anti COX2 (Abcam cat. ab15191) 1:200 dilution

*β-ACTIN*: mouse monoclonal anti β-actin (Abcam cat. ab8226) 1:2000 dilution)

**HRP-conjugates**

Goat anti rabbit IgG (H+L)-HRP conjugate (Bio-Rad cat.170-6515)

Goat anti mouse IgG(H+L)-HRP conjugate (Bio-Rad cat. 170-6516)

Rabbit anti goat F(AB)2 HRP XADs (NOVEX cat. A24452)

**List of secondary Antibodies**

*SOX-9*: rabbit polyclonal anti sox9 (Abcam cat. ab71762) 1:200 was matched with goat anti-rabbit IgG conjugate

*HAPLN1(K-14):* goat polyclonal anti HAPLN1(K-14) (Santa Cruz Biotechnology cat. sc-46862) 1:500 dilution was matched with Rabbit anti goat F(AB)2 HRP XADs (NOVEX cat. A24452)

*FIBROMODULIN*: rabbit polyclonal anti-Fibromodulin (NOVUS cat. NBP216494) 1:200 dilution was matched with Goat anti rabbit IgG (H+L)-HRP conjugate (Bio-Rad cat.170-6515)

*MMP3*: rabbit monoclonal anti MMP3 (Abcam cat. ab52915) 1:200 dilution was matched with Goat anti rabbit IgG (H+L)-HRP conjugate (Bio-Rad cat.170-6515)

*MMP13*: rabbit polyclonal anti MMP13 (Abcam cat. ab39012) 1:200 dilution was matched with Goat anti rabbit IgG (H+L)-HRP conjugate (Bio-Rad cat.170-6515)

COX2: rabbit polyclonal anti COX2 (Abcam cat. ab15191) 1:200 dilution was matched with rabbit polyclonal anti COX2 (Abcam cat. ab15191) 1:200 dilution

*β-ACTIN*: mouse monoclonal anti β-actin (Abcam cat. ab8226) 1:2000 dilution) was matched with Goat anti mouse IgG(H+L)-HRP conjugate (Bio-Rad cat. 170-6516)
